# The Relationship between physical activity, nutritional status, and sarcopenia in community- dwelling older adults with type 2 diabetes: a cross-sectional study

**DOI:** 10.1186/s12877-024-05038-6

**Published:** 2024-06-07

**Authors:** Chun-hui Ji, Xiao-qin Huang, Yue Li, Aikeremujiang Muheremu, Zhao-hui Luo, Zheng-hui Dong

**Affiliations:** 1 Zhejiang Hospital, Hangzhou, 310030 China; 2 The Third People’s Hospital of Chengdu, Chengdu, 610014 China; 3 The Sixth Affiliated Hospital of Xinjiang Medical University, Urumqi, 830002 China

**Keywords:** Sarcopenia, Type 2 diabetes mellitus (T2DM), Mini Nutrition Scale (MNA), International Physical Activity Short Questionnaire (IPASQ), Metabolic Equivalent of Task (MET)

## Abstract

**Aim:**

This study was conducted in Urumqi, Xinjiang, to assess the prevalence of sarcopenia and to determine the relationship between physical activity, nutritional status, and sarcopenia among community-dwelling patients with type 2 diabetes mellitus.

**Methods:**

Four hundred eight cases of older people patients with type 2 diabetes mellitus in the community in Urumqi, Xinjiang, from May to August 2022 were selected for a cross-sectional on-site survey, and general information questionnaires, clinical information surveys, physical function measurements, and criteria developed by the Asian sarcopenia working group in 2019 were selected for diagnosis of sarcopenia, and unifactorial and multifactorial binary Logistic regression were applied to analyze the influencing factors of T2DM combined with sarcopenia in patients with sarcopenia.

**Results:**

Among the 408 patients, 84 (20.6%) had sarcopenia, with a prevalence of 12.6%, 32.1%, and 51.9% in those aged 60–70, 71– 80, and 81 or older respectively. The prevalence increased significantly with age. Adjusting for variables, the study found that FFM of the Left Leg (OR: 0.710, 95% CI: 0.612–0.804, *P* = 0.024), FFM of the Right Arm (OR: 0.710, 95% *CI*: 0.612–0.804, *P* < 0.001), Age (OR: 1.246, 95% CI: 1.031–1.505, *P* = 0.023), Fasting Blood Glucose (OR: 1.649, 95% CI: 1.066–2.550, *P* = 0.025), and Post-Prandial Blood Glucose (OR: 1.455, 95% CI: 0.999–2.118, *P* = 0.025) were independent associated factors. An increase in MNA score (OR: 0.398, 95% CI: 0.244–0.6500, *P* < 0.001), ASMI (OR: 0.000, 95% CI: 0.00–0.01, *P* < 0.001) walking energy expenditure (MET-min) (OR: 0.998, 95% CI: 0.996–0.999, *P* = 0.001) reduced the prevalence of sarcopenia.

**Conclusion:**

This study shows that increased age, increased skeletal muscle mass index, decreased right arm FFM, increased postprandial glucose, increased MNA scores, and increased walking energy expenditure (MET-min) were associated with type 2 diabetes with sarcopenia.

## Introduction

The global aging population is a growing concern, with the number of people over 60 expected to double by 2050 [1]. According to the prediction of the International Diabetes Federation, global diabetes mellitus patients will reach 783.2 million in 2045 [2].The occurrence of type 2 diabetes mellitus in China will be the highest in the world. The persistent high occurrence of type 2 diabetes mellitus imposes a significant economic burden, causing to increased hospitalization and the risk of medical costs and death among the older people. Diabetes complications seriously affect the quality of life of older people patients [3]. The prevalence of insulin resistance leading to type 2 diabetes mellitus is increasing in Asia, especially in areas with aging populations [4]. Sarcopenia is a progressive and widespread age-related skeletal muscle disorder that increases the risk of disability, falls, and fall related injuries and increases hospitalization and mortality rates for patients [5]. The global pandemic of sarcopenia, skeletal muscle loss, and weakness, which prevails in up to 50% of older adults, is increasing worldwide due to the expansion of aging populations (especially if they are older than 80, frail and inactive), there is a strong relationship between nutrition [6], exercise [7] and sarcopenia. The MNA is a Mini Nutrition Scale specifically designed to evaluate the nutritional status of older adults, which includes anthropometric measurements, a holistic assessment, a dietary questionnaire, and a subjective assessment [8]. However, the relationship between MNA and IPAQ in type 2 diabetes mellitus with sarcopenia is unclear. Therefore, the aim of this study was to explore the association of MNA and IPAQ with type 2 diabetes mellitus with sarcopenia.

## Method

### Study participants

A total of 408 older people residents with T2DM in the Urumqi, Xinjiang community were selected. The inclusion criteria:①age ≥ 60 years old;②in line with the diagnosis of type 2 diabetes [9];③clear study purpose and active cooperation;④normal renal function; exclusion criteria:①with swallowing disorders, gastrointestinal tract dysfunction;②audiovisual as well as cognitive impairment; ③heart disease; ④taking nutritional supplements; ⑤Parkinson's disease, motor neuron disease, post-stroke, cerebral infarction, etc. affecting activity function; ⑥with other parts of the tumor ⑦suffer from Alzheimer's disease or serious mental illness; ⑧have obvious disabilities, need assisted walking, cannot participate in grip strength, gait speed test; ⑨acute changes in body composition (such as dehydration, edema); do not agree to participate in the project.

### Data collection

Data on diabetes duration, cumulative sleep duration, nutritional status, and physical activity level were collected using standardized questionnaires and assessments.

### Definition of sarcopenia

Sarcopenia was defined using internationally recognized physical tests: [9–11] (1) Muscle content, using the Inbody 120 body composition analyzer (polyconductance bioelectrical impedance principle). (2) Muscle strength was measured by applying the SPT- M276328 grip strength tester, and the maximum value was taken from the left and right hands twice after zero calibration. (3) Somatic function and six-meter step speed were measured, and then walking speed was calculated as m/s; five sit-to-stand tests were performed. And diagnostic criteria following the 2019 AWGS2 guidelines [10]. :① muscle content: ASM/height 2 (kg/m^2^) ≤ 7.0 (male) or ≤ 5.7(female) as reduced; ②muscle strength: grip strength ≤ 28 kg (male) or ≤ 18 kg (female) as reduced; ③somatic function: 6 m step speed ≤ 1.0 m/s and five sit-to-stand tests ≥ 12 s as reduced motor function. Those who met item① were pre-muscular hyposmia; those who met item ①② or ①③ were muscular hyposmia; and those who met item ①②③ were severe muscular hyposmia. According to the diagnostic criteria and staging, item ①② was included in this study.

### Nutritional surveys

The source scale MNA is a mini-nutrition scale developed specifically to evaluate the nutritional status of the older people, created by Guigoz et al. [8] in 1996. It has a sensitivity of 0.96, specificity of 0.98 and accuracy of 0.97 [12]. It takes only 10 min to complete and does not require laboratory tests. It includes anthropometric measurements, overall assessment, dietary questionnaire and subjective assessment, with a total of 18 indicators and a total score of 30 points. If the MNA value > 24, grade A, good nutritional status; MNA value 17.0–23.5, grade B, potential malnutrition; MNA value < 17, grade C, malnutrition.

### Physical activity survey

The International Physical Activity Short Questionnaire (IPAQ-S) was used to assess the physical activity of patients in the past 7 days. It consists of 7 items, divided into 4 categories: heavy physical activity, moderate physical activity, walking, and sitting [13]. Short rolls only simply asked about different intensities of activity by walking, moderate intensity and high intensity 1-week frequency and cumulative time per day, but across intensities the survey. The respondents were still required to combine the four categories of physical activity mentioned above. IPAQ MET was assigned a value of 3.3 for walking, 4.0 for moderate-intensity activity, and 4.0 for high-intensity activity. For the IPAQ short form, the weekly physical activity level of an individual engaged in a certain intensity is: the MET value corresponding to the physical activity × weekly frequency (d/w) × time per day (min/d), and the sum of the three intensity levels of physical activity is the total physical activity level.

### Statistical analyses

Data were expressed as means (standard deviation [14]) or frequencies of potential confounding variables. Participants were divided into two groups based on their MNA and IPAQ scores. Differences in continuous and categorical variables were evaluated using the Student's t-test and chi-squared test, respectively. Correlations were analyzed using Pearson's correlation coefficient. Logistic regression analyses were conducted to obtain the odds ratio (OR) and 95% confidence interval (CI) for MNA and IPAQ on the prevalence of sarcopenia after adjusting for demographic information such as age and gender. Statistical analysis was performed using SPSS (SPSS, Chicago, IL). P values of < 0.05 were considered significant.

## Results

In the study of 408 patients with type 2 diabetes, 84 (20.6%) were found to have sarcopenia. The prevalence of comorbid sarcopenia was 12.6%, 32.1%, and 51.9% in patients aged 60–70, 71–80, and 81 years or older, respectively. There was a significant increase in prevalence with advancing age. There were no significant differences between sarcopenia and non-sarcopenia in terms of gender, race, residency status, systolic blood pressure, diastolic blood pressure levels, body fat, body fat percentage, right upper extremity fat mass, right lower extremity fat mass, left lower extremity fat mass, InBody score, waist-to-hip ratio, and obesity level (*P* > 0.05). However, comparisons between patients with sarcopenia and non-sarcopenic patients revealed statistically significant differences in age group, body mass index (BMI = weight (kg)/height (m)2), education, and monthly income. Additionally, fasting blood glucose, postprandial blood glucose, duration of diabetes mellitus, somatic functioning (five-repetition sit-to-stand test, six-meter step speed), and sedentary time were significantly higher in sarcopenic patients than in non-sarcopenic patients. Conversely, sleep time, waist-to-hip ratio, and obesity were significantly lower in sarcopenic patients than in non-sarcopenic patients. Furthermore, sleep duration, grip strength, basal metabolic rate, total body water, protein content, inorganic salt content, skeletal muscle content, limb muscle mass, and left upper limb fat mass were significantly lower in patients with sarcopenia than in the non-sarcopenia group (*P* < 0.05, *P* < 0.001, Table 1).

**Table 1:** Clinical characteristics of study participants

	N	Type 2 diabetes mellitus with sarcopenia		*χ*^*2*^*/t*	*P*	
		No [*N* = 324(79.4%)]	Yes [*N* = 84(20.6%)]			
Sex (men/women)						
Men	201	155(77.1%)	46(22.9%)	1.279	0.258	
Women	207	169(81.6%)	38(18.4%)			
Age(years)						
60–70	269	235(87.4%)	34(12.6%)	35.683	< 0.001	
71–80	112	76(67.9%)	36(32.1%)			
> 81	27	13(48.1%)	14(51.9%)			
BMI(Kg/m ^2^)						
< 18.5	10	3(30%)	7(70%)	71.647	< 0.001	
18.5 ~ 23.9	145	88(60.7%)	57(39.3%)			
24 ~ 27.9	184	167(90.8%)	17(9.2%)			
≥ 28	69	66(95.7%)	3(4.3%)			
Education level						
Primary School	102	82(80.4%)	20(19.6%)	15.587	0.001	
Middle School	141	106(75.2%)	35(24.8%)			
High School	89	64(71.9%)	25(28.1%)			
University	76	72(94.7%)	4(5.3%)			
Nationalities						
Han people	363	285(78.5%)	78(21.5%)	4.279	0.370	
Uighur people	15	11(73.3%)	4(26.7%)			
Hui people	24	22(91.7%)	2(8.3%)			
Kazakh people	4	4(100%)	0(0)			
Other	2	2(100%)	0(0)			
Monthly income(yuan)				31.221	< 0.001	
< 1000	40	24(60%)	16(40%)			
1000–3000	82	54(65.9%)	28(34.1%)			
3001–5000	191	169(88.5%)	22(11.5%)			
5000–10000	83	65(78.3%)	18(21.7%)			
> 10,000	12	12(100%)	0(0)			
Residence situation						
Living with family	355	281(79.2%)	74(20.8%)	0.110	0.740	
Living alone	53	43(81.1%)	10(18.9%)			
SBP (mmHg)	408	129.43 ± 13.764	125.73 ± 15.936	2.834^b^	0.093	
DBP (mmHg)	408	77.72 ± 9.078	75.29 ± 9.220	1.043^b^	0.308	
Fasting blood sugar(*mmol/L*)	408	7.54 ± 2.404	9.30 ± 1.904	6.993^b^	0.009	
Post-prandial blood glucose(*mmol/L*)	408	10.61 ± 2.278	11.20 ± 2.750	9.873^b^	0.002	
Duration of diabetes(years)	408	6.69 ± 5.036	9.59 ± 7.719	42.148^b^	< 0.001	
Meditation time(s)	408	1.91 ± 1.283	2.93 ± 1.585	7.663^a^	0.006	
Sleep time(h)	408	7.46 ± 1.456	6.93 ± 0.991	8.950^a^	0.003	
		Type 2 diabetes mellitus with sarcopenia		*t*		*P*
		No[324例 (79.4%)])	Yes[84例 (20.6%)]			
ASMI		7.47 ± 0.92	6.05 ± 0.62	22.913		< 0.001
Grip strength (left)		25.60 ± 7.316	18.48 ± 5.555	6.295		0.012
Grip strength (right)		25.47 ± 7.913	19.68 ± 5.757	7.695		0.006
Five sit-to-stand tests		12.45 ± 2.977	16.94 ± 4.818	41.075		< 0.001
Six-meter stride speed		7.60 ± 2.506	11.54 ± 5.070	124.868		< 0.001
BMR (Basal Metabolic Rate)		1447.61 ± 194.181	1271.86 ± 1600.672	13.073		< 0.001
TBW (Total Body Water)		36.8 ± 6.638	31.02 ± 4.819	19.194		< 0.001
Protein		9.78 ± 1.781	8.14 ± 1.475	13.096		< 0.001
Minerals		3.33 ± 0.589	2.84 ± 0.497	10.725		0.001
BFM (Body Fat Mass)		20.72 ± 6.761	15.79 ± 5.782	1.051		0.306
SMM (Skeletal Muscle Mass)		27.52 ± 5.369	22.58 ± 4.465	12.654		< 0.001
PBF (Percent Body Fat)		29.03 ± 7.333	26.68 ± 8.218	0.71		0.4
FFM of Right Arm		2.84 ± 0.672	2.22 ± 0.506	12.658		< 0.001
FFM of Left Arm		2.83 ± 0.676	2.17 ± 0.481	19.545		< 0.001
FFM of Right Leg		7.43 ± 1.474	5.81 ± 0.957	30.042		< 0.001
FFM of Left Leg		7.4 ± 1.489	5.83 ± 0.987	30.487		< 0.001
BFM of Right Arm		1.39 ± 0.72	1.03 ± 0.487	3.77		0.053
BFM of Left Arm		1.4 ± 0.713	1.05 ± 0.484	4.395		0.037
BFM of Right Leg		2.91 ± 0.833	2.3 ± 0.753	1.787		0.182
BFM of Left Leg		2.89 ± 0.836	2.3 ± 0.752	1.86		0.173
WHR (Waist-Hip Ratio)		0.91 ± 0.056	0.86 ± 0.079	2.665		0.103
Obesity Degree		120.22 ± 17.236	101.68 ± 17.601	0.191		0.663

*SBP* Systolic blood pressure, *DBP* Diastolic blood pressure, *BFM* Body Fat Mass, *PBF* Percent Body Fat, *WHR* Waist-Hip Ratio, *SMI* skeletal muscle mass index, *BMR* Basal Metabolic Rate, *TBW* Total Body Water, *SMM* Skeletal Muscle Mass

^a^Represents t'-test result, ^b^Represents t-test result

Both MNA and IPAQ were found to be correlated with various body composition measures including total body water, protein, minerals, body fat mass (BFM), skeletal muscle mass (SMM), body mass index (BMI), fat-free mass (FFM) in different body parts, basal metabolic rate (BMR), waist-to-hip ratio (WHR), obesity level, and appendicular skeletal muscle mass index (ASMI) (Table 2).

**Table 2:** Comparison of the correlation between MNA and IPAQ and body composition in two groups of patients, respectively

MNA			IPAQ		
	*r*值	*P*值		*r*值	*P*值
MNA Total Score	1.000		Total physical activity in 1 week (MET-min)	1.000	
sarcopenia	-0.635**	< 0.01	sarcopenia	-0.593**	< 0.01
TBW (Total Body Water)	0.277**	< 0.01	TBW (Total Body Water)	0.277**	< 0.01
Protein	0.286**	< 0.01	Protein	0.286**	< 0.01
Minerals	0.270**	< 0.01	Minerals	0.272**	< 0.01
BFM (Body Fat Mass)	0.348**	< 0.01	BFM (Body Fat Mass)	0.145**	0.003
SMM (Skeletal Muscle Mass)	0.286**	< 0.01	SMM (Skeletal Muscle Mass)	0.284**	< 0.01
BMI (Body Mass Index)	0.426**	< 0.01	BMI (Body Mass Index)	0.268**	< 0.01
PBF (Percent Body Fat)	0.191**	< 0.01	PBF (Percent Body Fat)	0.000	0.992
FFM of Right Arm	0.310**	< 0.01	FFM of Right Arm	0.295**	< 0.01
FFM of Left Arm	0.298**	< 0.01	FFM of Left Arm	0.302**	< 0.01
FFM of Right Leg	0.293**	< 0.01	FFM of Right Leg	0.312**	< 0.01
FFM of Left Leg	0.289**	< 0.01	FFM of Left Leg	0.311**	< 0.01
BFM of Right Arm	0.292**	< 0.01	BFM of Right Arm	0.089	0.071
BFM of Left Arm	0.293**	< 0.01	BFM of Left Arm	0.080	0.107
BFM of Trunk	0.355**	< 0.01	BFM of Trunk	0.168**	0.001
BFM of Right Leg	0.343**	< 0.01	BFM of Right Leg	0.134**	0.007
BFM of Left Leg	0.333**	< 0.01	BFM of Left Leg	0.125*	0.012
InBody Score	0.142**	0.04	InBody Score	0.195**	< 0.01
BMR (Basal Metabolic Rate)	0.278**	< 0.01	BMR (Basal Metabolic Rate)	0.278**	< 0.01
WHR (Waist-Hip Ratio)	0.329**	< 0.01	WHR (Waist-Hip Ratio)	0.196**	< 0.01
Obesity Degree	0.445**	< 0.01	Obesity Degree	0.255**	< 0.01
ASMI	0.405**	< 0.01	ASMI	0.363**	< 0.01

Patients diagnosed with sarcopenia exhibited significantly lower values in anthropometric assessment, comprehensive assessment, dietary assessment, subjective assessment, total sarcopenia score, walking energy expenditure (MET-min), walking days, walking time, and sedentary time compared to patients without sarcopenia (Table 3).

**Table 3:** Micro nutritional scale and international physical activity scale according to whether type 2 diabetes is combined with sarcopenia

	Type 2 diabetes mellitus with sarcopenia		*χ*^*2*^*/t*	*P*
MNA(Scores)	No[324例 (79.4%)])	Yes[84例 (20.6%)]		
Anthropometric evaluation	6.799 ± 1.249	5.720 ± 1.568	5.844^a^	< 0.001
Global evaluation	8.512 ± 0.501	8.381 ± 0.488	2.185^a^	0.031
Dietetic evaluation	6.157 ± 0.916	5.720 ± 0.746	4.040^b^	< 0.001
Subjective assessment	3.719 ± 0.675	2.238 ± 0.889	14.226^a^	< 0.001
MNA Total Score	25.1883 ± 2.067	22.0595 ± 1.658	12.837^b^	< 0.001
MNA level				
Normal nutrition	271	7	174.470	< 0.001
Risk of malnutrition	51	75		
Dystrophy	2	2		
IPAQ				
Walking energy expenditure(MET-min)	2241.35 ± 1332.421	625.43 ± 625.440	15.735^b^	< 0.001
Number of walking days (d)	6.15 ± 0.722	3.76 ± 0.965	21.159^b^	< 0.001
Walking time(min)	111.11 ± 64.405	48.33 ± 41.387	10.896^b^	< 0.001
Meditation time (min)	114.66 ± 79.547	176.07 ± 95.072	5.447^b^	< 0.001

^a^Represents t'-test result, ^b^Represents t-test result

Age, gender, education level, monthly income, fasting blood sugar, sedentary time, duration of diabetes, sleep time, somatic function index, body composition index and other related factors were included in the binary logistic regression model. The prevalence increased significantly with age. adjusting the variables reveals, it was found that FFM of the Left Leg (OR: 0.511, 95% CI: 0.342–0.869, *P* = 0.024), FFM of the Right Arm (OR: 0.710,95% *CI*: 0.612–0.804, *P* < 0.001), Age (OR: 1.246,95% *CI*: 1.031–1.505, *P* = 0.023), ASMI (OR: 0.000, 95% *CI*: 0.00–0.01, *P* < 0.001), Fasting blood glucose (OR: 1.649,95% CI: 1.066–2.550, *P* = 0.025), and Post-prandial blood glucose (OR: 1.455,95% CI: 0.999–2.118, *P* = 0.025) were independent associated factors. The prevalence of sarcopenia was decreased by a moderate increase in MNA score (OR: 0.398, 95% CI: 0.244–0.6500, *P* < 0.001) and walking energy expenditure (MET-min) (OR: 0.998, 95% CI: 0.996–0.999, *P* = 0.001). (Table 4).

**Table 4:** Odds ratio of the MNA and IPAQ on the presence of sarcopenia

	Model 1		Model 2		Model 3		Model 4		Model 5	
	OR(95%*CI*)	P	OR(95%*CI*)	P	OR(95%*CI*)	P	OR(95%*CI*)	P	OR(95%*CI*)	*P*
Age	-	-	-	-	-	-	1.159(1.018–1.319)	0.026	1.246(1.031–1.505)	0.023
ASMI	-	-	0.001(0.000–0.007)	< 0.001	0.000(0.00–0.10)	< 0.001	0.000(0.00–0.02)	< 0.001	0.000(0.00–0.01)	< 0.001
FFM of Right Arm	-	-	0.603(0.521–0.818)	< 0.001	0.579(0.486–0.820)	< 0.001	0.629(0.574–0.758)	< 0.001	0.710(0.612–0.804)	< 0.001
FFM of Left Leg	-	-	-	-	-	-	0.245(0.228–0.410)	0.028	0.511(0.342–0.869)	0.024
Education level	-	-	-	-	-	-	-	-	3.026(0.753–12.157)	0.119
Fasting blood glucose	-	-	-	-	-	-	-	-	1.649(1.066–2.550)	0.025
Post-prandial blood glucose	-	-	-	-	1.369(1.050–1.785)	0.02	1.386(1.024–1.876)	0.035	1.455(0.999–2.118)	0.05
MNA score	0.378(0.307–0.467)	< 0.001	0.411(0.315–0.537)		0.404(0.280–0.583)	< 0.001	0.389(0.254–0.596)	< 0.001	0.398(0.244–0.6500	< 0.001
Walking energy expenditure(MET-min)	-	-	-	-	0.998(0.997–0.999)	< 0.001	0.998(0.997–0.999)	0.003	0.998(0.996–0.999)	0.001

Model 1 is unadjusted; Model 2 is adjusted for ASMI, FFM of Right Arm; Model 3 is adjusted for ASMI, FFM of Right Arm, Post-prandial blood glucose, Walking energy expenditure(MET-min); Model 4 is adjusted for ASMI, FFM of Right Arm, Post-prandial blood glucose, Walking energy expenditure(MET-min),Age, FFM of Left Leg; Model 5 is adjusted for ASMI, FFM of Right Arm, Post-prandial blood glucose, Walking energy expenditure(MET-min),Age, FFM of Left Leg; Education level, Fasting blood glucose

## Discussion

### Sarcopenia and age in T2DM

Sarcopenia in patients with T2DM was significantly associated with age. A cross-sectional study by Cui et al. [15] found that the prevalence of sarcopenia was 28.8% among older adults with type 2 diabetes mellitus aged 65 years or older in Changchun City, and Shariff-Ghazali Sazlina et al. [16] found that 28.5% of older adults with T2DM (aged 60 years or older) attending a public primary healthcare clinic in Malaysia had sarcopenia. In this study, the detection rate of type 2 diabetes mellitus combined with sarcopenia was found to be 20.6% in the Urumqi community in Xinjiang. The prevalence of comorbid sarcopenia was 12.6%, 32.1%, and 51.9% in patients aged 60–70, 71–80, and 81 years or older, respectively. In fact, according to Cui et al. [15], the prevalence of T2DM sarcopenia gradually increases with age (17.4% in the 65–69 years age group, 28.1% in the 70–74 years age group, 52.4% in the 75–80 years age group, and 60% in the age group of 80 years or older). The results are slightly different due to the stratification of the study. However, it can also be concluded that the prevalence of sarcopenia in patients with type 2 diabetes mellitus increases with age, as well as the risk of developing sarcopenia.

### Sarcopenia and Indicators Related to Muscle Mass in T2DM

This study found that low muscle mass in the lower extremities was a common problem in patients with T2DM combined with sarcopenia. AWGS 2019 also clearly stated that low grip strength, slow gait speed and low SMI were diagnostic criteria for sarcopenia. Chia-Ling Lin et al. studied the association between body composition and type 2 diabetes and found [17] that body weight, BFM, FFM, ASM and SMI all decreased with age. Meanwhile another study found [17] that lower muscle mass standards than those associated with chronic diseases such as diabetes, especially lower limb muscle mass led to a significantly increased risk of developing diabetes. Recent studies have shown that lower limb skeletal muscle mass in T2DM patients is associated with poor metabolic control. And the present study also found that patients with T2DM had a low basal metabolic rate. The results of the present study are all consistent with the findings of the above studies. Although it is an unavoidable fact that muscle mass decreases with age, it has still been found [18] that types of exercise interventions such as exercises targeting resistance (strength and explosive power), aerobic, balance and flexibility exercises can improve physical function and may reduce blood glucose levels, decrease blood glucose fluctuations, and reduce the risk of hypoglycemia in older patients with type 2 diabetes [19]. Therefore, the development of effective and feasible exercise modalities can delay the adverse outcome of sarcopenia in T2DM.

### Sarcopenia and indicators related to diabetes in T2DM

This study found that fasting blood glucose and postprandial blood glucose were significantly different in the group of people with type 2 diabetes who developed sarcopenia versus those who did not, and that elevated postprandial blood glucose increased the risk of developing sarcopenia in combination with T2DM. A meta-study found that [20], the prevalence of sarcopenia in type 2 diabetes is high, especially in patients with poor glycemic control. A cross-sectional study in Changchun found [15]. Fasting blood glucose, 2-h postprandial blood glucose, and glycosylated hemoglobin, were not significantly different between the sarcopenia and non-sarcopenia groups. Therefore, there is controversy regarding the effects of fasting blood glucose and postprandial blood glucose on sarcopenia, and further optimization of the study design and close monitoring of blood glucose changes in the study population are needed.

### Sarcopenia and MNA in T2DM

Currently, there is a little evidence of the interrelationship between nutritional status, T2DM and sarcopenia in older people individuals. In this study, we found that there was a difference between sarcopenia and non-sarcopenia in the type 2 diabetes population in terms of MNA scores and that an increased total MNA assessment score was a protective factor for sarcopenia in type 2 diabetes. Shun Matsuura et al. found [21] that malnutrition was associated with a high risk of sarcopenia in older patients and that low GNRI was associated with an increased risk of sarcopenia in older patients with type 2 diabetes. A cross-sectional study of male nursing home residents in Turkey found [22], Sarcopenic residents had lower MNA score than non-sarcopenic residents FFM was significantly lower in the residents with malnutrition compared to well-nourished residents. In a cross-sectional study in Mexican nursing homes, María C Velázquez-Alva et al. found [6] that Women with a poor nutritional status were more likely to have sarcopenia (*OR*: 4.97, *P* = 0.003) whilst those with T2DM showed a higher probability of sarcopenia (*OR*: 5.52, *P* = 0.019) than women without T2DM. Sarcopenia was very common in women with poor nutritional status and T2DM.

### Sarcopenia and IPAQ in T2DM

Through this investigation, it was found that there was a significant difference in the total IPAQ MET scores between the sarcopenic and non-sarcopenic groups of patients with type 2 diabetes mellitus, which was mainly attributed to the lack of proper professional guidance for physical activity at high and moderate physical activity intensities as well as poor glycemic control and the presence of fear in the patients, which was more pronounced in the sarcopenic group. In a recent longitudinal study on the progression of muscle status in community-dwelling ambulatory older multiracial Asian patients with type 2 diabetes mellitus [23], 26. 6% were less physically active, mainly due to prolonged time spent at home as a result of official coercive measures such as segregation, quarantine, and social distancing during the current COVID-19 pandemic, so that home-based exercise is discouraged during the pandemic Sedentary and physical inactivity in older adults is a potential solution, especially for the T2DM patients in the study [23]. Appropriate home physical activity and exercise may support not only their muscular health, but also their glycemic control and psychological well-being.

## Conclusion

This study suggests that factors such as nutritional status, physical activity level Glucose control are associated with the presence of sarcopenia in patients with T2DM combined with sarcopenia.

## Data Availability

There are currently unpublished articles on this data, and data disclosure is not provided at this time; it will be available from the corresponding author upon reasonable request in the future.

## References

[1] Cho SJ, Stout-Delgado HW (2020). Aging and Lung Disease. Annu Rev Physiol.

[2] Wang R, Wang QY, Bai Y (2023). Research progress of diabetic retinopathy and gut microecology. Front Microbiol.

[3] Osaka T, Hamaguchi M, Hashimoto Y (2018). Decreased the creatinine to cystatin C ratio is a surrogate marker of sarcopenia in patients with type 2 diabetes. Diabetes Res Clin Pract.

[4] Ramachandran A, Snehalatha C, Shetty AS (2012). Trends in prevalence of diabetes in Asian countries. World J Diabetes.

[5] Cruz-Jentoft AJ, Sayer AA (2019). Sarcopenia. Lancet (London, England).

[6] Velázquez-Alva MC, Irigoyen-Camacho ME, Zepeda-Zepeda MA (2020). Sarcopenia, nutritional status and type 2 diabetes mellitus: A cross-sectional study in a group of Mexican women residing in a nursing home. Nutr Dietet.

[7] Kemp VL, Piber LS, Ribeiro AP (2021). Can physical activity levels and relationships with energy expenditure change the clinical aspects of sarcopenia and perceptions of falls among older people women? Observational cross-sectional study. Sao Paulo Med J=Revista paulista de medicina..

[8] Guigoz Y, Vellas B, Garry PJ (1996). Assessing the nutritional status of the older people: The Mini Nutritional Assessment as part of the geriatric evaluation. Nutr Rev.

[9] Chen LK, Woo J, Assantachai P (2020). Asian Working Group for Sarcopenia: 2019 consensus update on sarcopenia diagnosis and treatment. J Am Med Dir Assoc.

[10] Cruz-Jentoft AJ, Bahat G, Bauer J (2019). Sarcopenia: revised European consensus on definition and diagnosis. Age Ageing.

[11] Dent E, Morley JE, Cruz-Jentoft AJ (2018). International Clinical Practice Guidelines for Sarcopenia (ICFSR): Screening, Diagnosis and Management. J Nutr Health Aging.

[12] Slee A, Birch D, Stokoe D (2015). A comparison of the malnutrition screening tools, MUST, MNA and bioelectrical impedance assessment in frail older hospital patients. Clin Nutr (Edinburgh, Scotland).

[13] Qu N, Li K J Z l x b x z z Z l z. Study on the reliability and validity of international physical activity questionnaire (Chinese Vision, IPAQ). 2004, 25(3):265-8. 15200945

[14] Jyväkorpi SK, Ramel A, Strandberg TE (2021). The sarcopenia and physical frailty in older people: multi-component treatment strategies (SPRINTT) project: description and feasibility of a nutrition intervention in community-dwelling older Europeans. European Geriatr Med.

[15] Cui M, Gang X, Wang G (2020). A cross-sectional study: Associations between sarcopenia and clinical characteristics of patients with type 2 diabetes. Medicine.

[16] Sazlina SG, Lee PY, Chan YM (2020). The prevalence and factors associated with sarcopenia among community living older people with type 2 diabetes mellitus in primary care clinics in Malaysia. PLoS ONE.

[17] Lin CL, Yu NC, Wu HC, et al. Association of body composition with type 2 diabetes: a retrospective chart review study. Int J Environ Res Public Health. 2021;18(9):4421. 10.3390/ijerph18094421 PMC8122668 33919339

[18] Angulo J, El Assar M, Álvarez-Bustos A (2020). Physical activity and exercise: Strategies to manage frailty. Redox Biol.

[19] Zhao D, Shi W, Bi L (2022). Effect of short-term acute moderate-intensity resistance exercise on blood glucose in older patients with type 2 diabetes mellitus and sarcopenia. Geriatr Gerontol Int.

[20] Wu CN, Tien KJ (2020). The impact of antidiabetic agents on sarcopenia in type 2 diabetes: a literature review. J Diabetes Res.

[21] Takahashi F, Hashimoto Y, Kaji A, et al. Association between geriatric nutrition risk index and the presence of sarcopenia in people with type 2 diabetes mellitus: a cross-sectional study. Nutrients. 2021;13(11):3729. 10.3390/nu13113729 PMC8618310 34835985

[22] Bahat G, Saka B, Tufan F (2010). Prevalence of sarcopenia and its association with functional and nutritional status among male residents in a nursing home in Turkey. Aging Male.

[23] Tan NC, Sankari U, Ng CE (2022). Longitudinal study on the progression of muscle status among community-dwelling ambulatory older multiethnic Asians with type 2 diabetes mellitus. BMC Geriatr.

